# Bayesian network-based over-sampling method (BOSME) with application to indirect cost-sensitive learning

**DOI:** 10.1038/s41598-022-12682-8

**Published:** 2022-05-24

**Authors:** Rosario Delgado, J. David Núñez-González

**Affiliations:** 1 Department of Mathematics, Universitat Autònoma de Barcelona, Campus de la UAB, 08193 Cerdanyola del Vallès, Spain; 2 Department of Applied Mathematics, University of the Basque Country (UPV/EHU), 29 Otaola Av., 20600 Eibar, Spain

**Keywords:** Engineering, Mathematics and computing

## Abstract

Traditional supervised learning algorithms do not satisfactorily solve the classification problem on imbalanced data sets, since they tend to assign the majority class, to the detriment of the minority class classification. In this paper, we introduce the Bayesian network-based over-sampling method (BOSME), which is a new over-sampling methodology based on Bayesian networks. Over-sampling methods handle imbalanced data by generating synthetic minority instances, with the benefit that classifiers learned from a more balanced data set have a better ability to predict the minority class. What makes BOSME different is that it relies on a new approach, generating artificial instances of the minority class following the probability distribution of a Bayesian network that is learned from the original minority classes by likelihood maximization. We compare BOSME with the benchmark synthetic minority over-sampling technique (SMOTE) through a series of experiments in the context of *indirect cost-sensitive learning*, with some state-of-the-art classifiers and various data sets, showing statistical evidence in favor of BOSME, with respect to the expected (misclassification) cost.

## Introduction

In classification, an imbalanced data set is one with a skewed class distribution. We can assume we mean binary class data sets (otherwise non-minority classes can be merged into a single *majority* class), with a majority class (*negative*), and the minority class (*positive*) being generally the one we are most interested in predicting.

Imbalanced data sets are pervasive across a multitude of fields, making it difficult for machine learning algorithms to identify the minority cases. In fact, detecting instances belonging to the minority class is generally difficult, and the cost associated with misclassifying them (*false negative*) is often much higher than that of misclassifying an instance of the majority class (*false positive*). There are many real-world situations, such as spam detection, fraud identification, disease diagnosis, or vital prognosis, where misclassifying a positive class is clearly worse than misclassifying a negative class. For example, in^1^ the minority class is the death of the patient in the ICU, and the cost of a false negative error, corresponding to classifying a patient who is going to die as a survivor, implies failing to recognize the severity of the situation and includes postponing or ruling out treatments that could actually improve the patient’s life expectancy, revealing the seriousness of this error.

This simple example shows the practical inadequacy of classical cost-insensitive classification, which focuses on maximizing accuracy but does not take into account the costs associated with different types of classification errors. This is because, due to the disparity of the class distribution, the algorithms learned from the data set tend to assign the majority class, misclassifying the minority cases, but at the same time giving the false impression of high accuracy. That is, algorithms learned from an unbalanced data set are biased towards the majority class and fail to learn the underlying patterns that distinguish between classes, so they are prone to overfit the majority class.

To address this issue, we focus on probably the most common approach, which is *over-sampling*.

### Over-sampling

*Over-sampling* is a suitable methodology to modify the class variable distribution at a data-level stage (pre-processing), before the learning process, to address the problem of learning classifiers from an imbalanced data set. In fact, it consists of creating new synthetic cases of the minority class based on the available data, and then learning the classification algorithm from the enlarged and more balanced data set, instead of using the original one.

The most widely used over-sampling algorithm is SMOTE (synthetic minority over-sampling technique) which was proposed in 2002^2^ as an alternative to the standard random over-sampling, based on interpolation between neighboring cases of the minority class, and became a pioneer for the research community in imbalance classification. Since then, it has become a benchmark for preprocessing imbalanced data for the purpose of learning classifiers from it, and has proven successful in a variety of applications from several different domains. Due to its popularity, SMOTE is the most influential over-sampling algorithm.

SMOTE is designed to deal with continuous features, since it over-samples the minority class by taking each minority class instance and introducing artificial cases by choosing points along line segments connecting it with one of its (typically 5) nearest minority class neighbors in the feature space, and translates the same methodology to the categorical scenario, a methodology that makes no sense in this case, although it may (or may not) give good practical results. In fact, it generates the synthetic instances along the line segments joining neighbors of the *k* nearest neighbors in the minority class, where *k* is a hyper-parameter to be specified. More specifically, to generate a new synthetic instance, randomly selects one of two values of any categorial feature: the one corresponding to an instance and one of its neighbors (see details in^3^). Even works that generalize SMOTE to handle mixed data sets of categorical and continuous features have the same drawback. For example^2^, introduces SMOTE-NC (Synthetic Minority Over-sampling TEchnique-Nominal Continuous) which, as described there, uses the median of the standard deviations of the continuous features of the minority class to define a “distance” between instances that differ in categorical features. Aside from the fact that this makes it impossible for this method to work with categorical data sets that do not contain continuous features (which BOSME can, however), it clearly lacks theoretical justification for this technique, regardless of whether experimentally it can experimentally give good results, since it requires working with the concept of “distance” between values taken by categorical variables. That is, the idea behind SMOTE lacks justification, in our opinion, for categorical features, and this method in no way approximates the distribution of minority instances.

In spite of this, until 2018, the date of the publication of^3^, a large number of SMOTE-based extensions have been proposed in the specialized literature. And nowadays, SMOTE is still used as the main method of over-sampling. See for example^4^, where it is used in combination with a support vector data description SVDD model, or^5^, where the G-SMOTE algorithm used in classification is extended to regression tasks, being G-SMOTE a variant of SMOTE that allows the generation of synthetic instances in a geometric region around the selected instances instead of in the line segment that joins them. And work continues to find variants that somehow compensate for SMOTE’s weaknesses focusing, for example, on the definition of the neighborhood to generate new minority samples using the Euclidean distance (see^6^). However, some works critical of SMOTE have begun to appear recently in the same vein as ours. An example is^7^, where two imbalanced binary data classification methods based on diversity over-sampling by generative methods are proposed as an alternative, just as we propose BOSME.

Another approach to dealing with imbalanced data sets is *under-sampling*, which is just the opposite of *over-sampling*, meaning a removal of instances of the majority class. There is even an intermediate approach, called *hybrid-sampling*, which uses a combination of both. See, for example, the wrapper framework for applying under-sampling and over-sampling using SMOTE in^8^. Nevertheless, in this paper we will focus on *over-sampling*, since it avoids the loss of information that comes with deleting instances.

The objective of our work has been the introduction of a new general methodology of *over-sampling*, which represents a new paradigm, called Bayesian network-based over-sampling method (BOSME), which pre-processes any set of imbalanced data by augmenting it with new cases of the minority class, so that any type of classifier can be learned from the enlarged data set. More specifically, BOSME consists of randomly generating new instances of the minority class using a Bayesian network. This Bayesian network is a model for the probabilistic relationships between the features that is learned from the subset of instances in the original data set that belong to the minority class, with the criterion of maximizing the likelihood.

### Bayesian networks

Bayesian networks (BN) are graphical models representing the probabilistic relationships among variables affecting a phenomenon, which can be (and usually are) used for probabilistic inference. For a set of random variables $V=\{X_1,\ldots ,\,X_n\}$, a BN is a model that represents their joint probability distribution *P*, the graphical part of the model consisting of a *directed acyclic graph* (DAG), whose *n* nodes represent the random variables. The directed arcs among the nodes represent conditional dependencies (not necessarily causal) governed by the *Markov condition*, which we explain below.

Node *X* is a “parent” of node *Y* (and *Y* is a “child” of *X*) if there is a directed arc in the DAG from *X* to *Y*. We denote by *PA*(*Y*) the set of parents of *Y*. If $PA(Y)=\emptyset$ we say that *Y* is a *root* node. If there is a *path* from node *Z* to node *T* (that is, a concatenation of directed arcs connecting them), then we say that *T* is a “descendant” of *Z*; if a node has no descendants, we say that it is a “leaf”. What characterizes the BN is the **Markov condition**, which can be expressed as follows: *each variable in V is conditionally independent of any of its non-descendants conditioning to the state of all its parents*. Moreover, *P* can be expressed as the product of the conditional distributions of all nodes given the values of their parents, whenever these conditional distributions exist. This is what is known as **chain rule** and is formally expressed for discrete/categorical variables as follows:

$$\begin{aligned} P(X_1=x_1,\,\ldots ,\,X_n=x_n)=\prod _{i=1}^n P(X_i=x_i/PA(X_i)) \end{aligned}$$

for all the possible values (*instantiations*) $x_i$ for $X_i$, $i=1,\ldots ,\,n$ (see Neapolitan^9^). The chain rule is very useful because it allows to obtain the joint distribution of the variables from the conditional distributions of each node to its parents, and from the marginal distributions of the root nodes. The probability values of these conditional and marginal distributions are the parameters of the BN to be learned from data.

We adopt the *hill climbing greedy search-and-score-based* structure learning algorithm to learn the DAG, which is the structure of the BN. This algorithm explores the space of the directed acyclic graphs by single-arc addition, removal and reversals, to find the structure that maximizes the score function, taking advantage of the score decomposability to decrease its complexity and make it computationally feasible. For our purpose we choose the logarithm of the likelihood function (**logLik**) as score function to be maximized, since it is a measure of how well the model fits the actually observed data when the parameters are estimated by using the *maximum likelihood estimation* (MLE) method.

Once we have learned from data the BN that represents the probabilistic dependency relations between the variables of *V*, both the structure and the parameters, we can obtain samples of instances following the probability distribution *P* entailed by the BN. For that, we will use the **logic sampling** (LS) algorithm^10^, that generates instances from the network distribution by randomly selecting values for each node, weighted by the probability of that value occurring. Indeed, LS generates the values of a new instance starting from the root nodes, which are sampled from their marginal probability distributions. The nodes are traversed from the “roots” down to the “leaves”, so at each step the weighting probability is either the marginal or the conditional probability distribution entry for the sampled parent values: once the values for the root nodes have been generated, the values of their children in the DAG are sampled from their conditional distributions (conditional on the values already sampled from the parents), and so on, iteratively, until that all nodes have been visited and the values of the “leaf” nodes have been sampled, and with them, those of all the nodes, finishing the process. That the instances generated in this way follow the distribution of *P* is a consequence of the *chain rule*.

Its character as a graphic model given by the DAG, together with the Markov condition and the Chain rule, which allow obtaining the joint probability distribution of the model variables (and, therefore, any other probability) from the conditional probabilities of each node to its parents in the DAG, make this probabilistic model a really versatile, useful and unique model in the current landscape of machine learning models.

BOSME is original and different from the other *over-sampling* methods in that it generates the new cases from a model chosen using the likelihood criterion: the more likely the model is for cases of the minority class, the more representative of this class the cases artificially generated from the model will be, and thus allow a classifier learned from the enlarged data set to better discriminate the classes.

But how many cases of the minority class must be artificially generated? It depends on what is intended with it. We will make sense of this question and answer it in the context of *cost-sensitive learning*.

### Cost-sensitive approach to classification

*Cost-sensitive learning* is a subfield of *machine learning* that takes into account misclassification costs when learning a classifier. It is closely related to the study of classification in the scenario of imbalanced data sets, so they share techniques and procedures (see^11^). The aim of a cost-sensitive classifier is to minimize the expected cost of (mis)classification.

Cost-sensitive learning techniques can be categorized into two groups: *black box* and *transparent box* (see^12^), which coincide, respectively, with the data-level and the algorithm-level approaches referred in^13^. The second category includes methods that modify the original learning algorithm to take cost into account, which makes it necessary to have a deep understanding of the algorithm itself, and thus the methods are algorithm-dependent. In contrast, the first category (also known as *indirect methods*^14^) uses techniques as *sampling*, *relabeling* and *weighting* before the learning of the classifier, to modify the training data set in a pre-processing phase, with the aim of obtaining a desired class distribution based on the misclassification costs. In this paper we focus on the *sampling indirect methods* for cost-sensitive learning, what are shared with the imbalanced classification problem. SMOTE has been chosen as the over-sampling method for the preprocessing of imbalanced data sets in relation to cost-sensitive learning by different authors^8,15^. In this paper we propose to use BOSME alternatively.

Much effort has been devoted so far to the development of cost-sensitive decision tree learners, but much less to the development of cost-sensitive Bayesian networks. See the recent paper^16^, in which direct and indirect approaches to cost-sensitive learning of Bayesian networks were followed, and experimentally compared with a cost-sensitive decision tree learning algorithm, showing that they are better in terms of misclassification costs and accuracy. In^17^, the indirect approach has been applied to some state-of-the-art Bayesian network classifiers, which perform better than when derived from the original training data set, and in^18^ cost-sensitive Bayesian networks apply to rock burst prediction.

In^12^, the authors used *resampling* and *reject sampling* as cost-sensitive basic indirect methods, the former presenting the risk of severe overfitting, and the latter requiring the averaging of different classifiers to improve predictive performance, which might not be very good since *reject sampling* implies a reduction of cases in the data set. Compared to them, the approach we focus on, based on the use of an *over-sampling* method, is a methodology that avoids these two sources of poor performance: overfitting and data set reduction.

When dealing with an imbalanced data set in the *cost-sensitive approach* scenario, considering *over-sampling* as a *sampling indirect method* and the expected cost as the performance metric, we see as application of the Folk Theorem how from the misclassification costs, we can determine a priori how many cases we should artificially generate from the minority class, so that the classifier that maximizes accuracy with the enlarged data set is the same that minimizes the expected cost with the original. In fact, the Folk Theorem states that for that, the distribution of the class variable must be modified with a factor proportional to the costs of misclassification. In this way, we can transform any supervised learning problem with costs into a costless one suitable for applying any cost-insensitive classifier learning algorithm, simply by conveniently extending the data entering the learning process with an appropriate number of new artificial instances of the minority class.

The layout of the paper is as follows. In “Section Bayesian network-based over-sampling method (BOSME)” we introduce and describe BOSME, including the pseudo-algorithm that implements it. “Section Application: sampling indirect method for cost-sensitive learning” explains the use of the Folk Theorem to determine the number of new artificial cases that will be generated from the minority class by applying BOSME. “Section Experiments” describes the experimental phase to evaluate BOSME and compare it with other methods of *over-sampling* such as SMOTE, considered as benchmark, and ROSE (Random Over-Sampling Examples). The results of these experiments are given in “Section Results”, and we conclude with a few words in “Section Conclusion”. To lighten “Section Results”, we moved to the “Appendix” some auxiliary tables.

## Bayesian network-based over-sampling method (BOSME)

We introduce BOSME as a theoretically well-motivated over-sampling preprocessing technique that can be used for general data sets. That is, it can be applied both when the features are (or can be transformed into) categorical, when they are mixed (categorical and continuous), and when they are all continuous. The goal is to generate new artificial instances of the minority class, and this method consists of randomly generate them from the joint probability distribution entailed by a Bayesian network that is constructed as the probabilistic model for the dependency relationships between the features in the minority class setting, with the highest likelihood. This makes BOSME a new paradigm for *over-sampling* methods.

The Bayesian network is learned from the subset of the data set corresponding to the instances belonging to the minority class. While parameters learning is carried out following the *Maximum Likelihood Estimation*, structure learning is performed following a score-based structure learning algorithm with the logarithm of the likelihood function (*logLik*) as the score function. In the case of mixed categorical and continuous features, we assume that

- categorical nodes can only have other categorical nodes as parents,
- the distribution of continuous nodes is a conditional linear Gaussian, that is, conditional on any combination of values of the categorical parents, and on any value of the continuous parents, the distribution of a continuous node is Gaussian with a linear function of the values of the continuous parents as mean value.

If all the features are continuous, we assume that they follow a joint Gaussian distribution and each variable is normally distributed, being its mean a linear function of its parents, and having a common standard deviation.

The learned Bayesian network is a pseudo-optimal probabilistic model for the relationship between the features of the minority class, since it reaches a local maximum of the likelihood function. Since the likelihood function is a measure of the goodness of fit of a model to the set of instances for given unknown parameters, it sounds quite natural to randomly generate a sample of new synthetic instances for the minority class following the joint probability distribution of the features entailed by the learned Bayesian network. The intuition of this method is clear, leaving aside its efficacy as an over-sampling method, which we will evaluate in the experimentation section, contrary to what happens with the benchmark over-sampling method SMOTE.

We introduce some notations: denote by *S* the original (imbalanced) data set, with *M* instances and with binary class variable *V*. Let $m_+$ be the number of instances corresponding to the minority (positive) class in *S*, and $m_-$ be the number of instances in the majority class, that is, $m_-=M-m_+$. We assume that $\frac{m_+}{M}<0.5$ (usually, but not necessarily, $<<0.5$). The original distribution of the class variable *V* in the data set *S* is therefore $(p_+,\,p_-)$, where $p_+=\frac{m_+}{M}$ and $p_-=\frac{m_-}{M}$. Therefore, BOSME’s goal is to generate a number of new instances, say *n*, of the minority class, such that in the enlarged data set augmented with the synthetically generated instances, denoted by ${\widetilde{S}}$, the minority class represents a desired proportion *q* of the total.

For the sake of explanation, we want to determine the number of instances that the over-sampling method will generate, *n*, such that

$$\begin{aligned} \frac{m_++n}{M+n}= q \,. \end{aligned}$$

Isolating from this equation, we get that

1 $$\begin{aligned} n&=round\Big (\,\frac{q\,M-m_+}{1-q}\,\Big ) \end{aligned}$$

rounding to the nearest integer, since *n* must be a (positive) integer. In this way, the proportion actually achieved will be approximately *q*.

**Figure 1:**
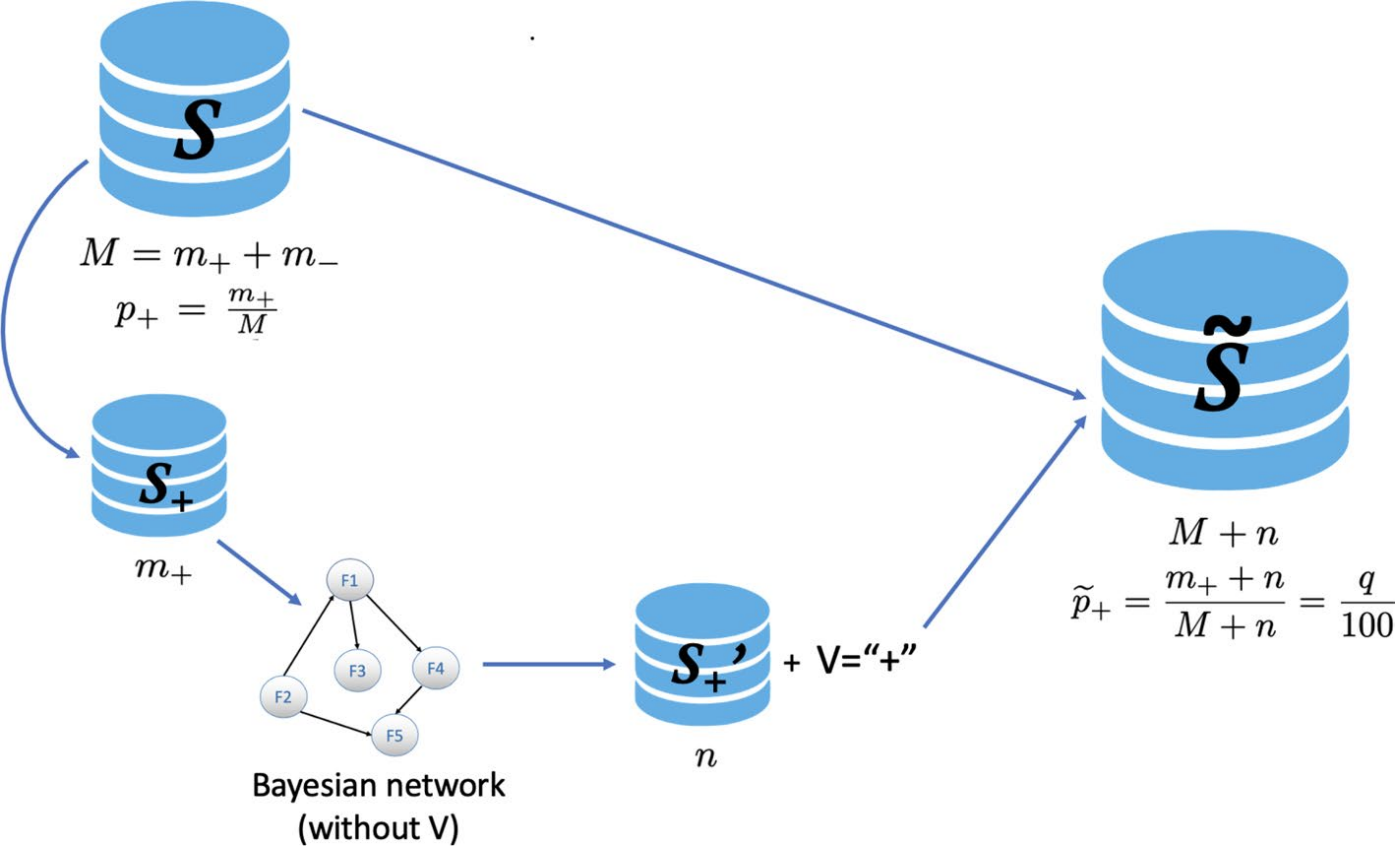
Graphical scheme of the BOSME algorithm.


The steps of the BOSME over-sampling method are detailed below (see Fig. 1 and the pseudo-code in Algorithm 1):

Step 1: Extract from the data set *S* the subset of the minority class, that is, the cases for which *V* is the positive class “+”, and denote it with $S_{+}$, which is composed of $m_+$ instances. Only if it makes sense, that is, if the proportion we would like for the minority class is greater than what it initially represents and less than 1 ($\frac{m_+}{M}<q<1$), we can continue.
Step 2: Construct a Bayesian network named BN as a model for the relationship between the model features (all variables except the class variable *V*) from the data set $S_+$, using a score-based structure learning algorithm with score the log-likelihood function (*logLik*). In this sense, BN is a pseudo-optimal model that (locally) maximizes the probability of the observed instances of the minority class.
Step 3: Simulate from BN as many new instances as needed by using the LS algorithm, to reach the desired *n* given by (1), with no missing values, forming a set of complete instances indicated by $S'_{+}$. Note that the class variable *V* does not appear in the generated synthetic instances, and must be added manually, taking the value of the minority (positive) class.
Step 4: Bind the synthetically generated instances corresponding to the minority class, $S'_{+}$, and the original *S*, to obtain the new enlarged data set ${\widetilde{S}}=S\cup S'_{+}$.

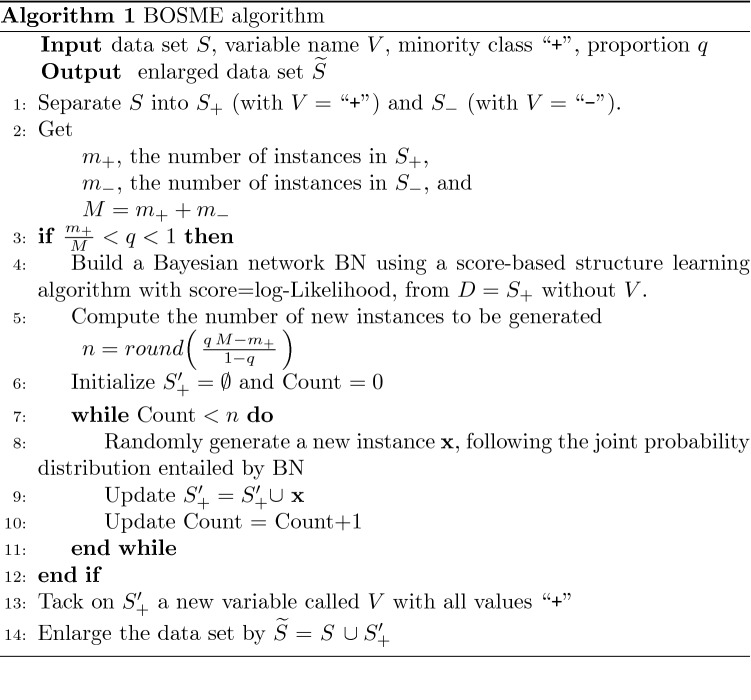


Note that the amount of over-sampling, *n*, is a parameter of the algorithm that is deduced by (1) from the input $q\in (\frac{m_+}{M},\,1)$, which is the desired proportion for the over-sampled minority class in the final data set, including the new synthetic instances.

## Application: sampling indirect method for cost-sensitive learning

We denote by $c_+$ and $c_-$, respectively, the cost associated with misclassifying instances belonging to the positive (*false negative*) and the negative (*false positive*) classes. We assume that $c_+>c_-$. So, if $\gamma =\frac{c_+}{c_-}$ denotes the **cost rate**, we assume that $\gamma >1$.

We use a Folk Theorem (*Translation Theorem 2.1*^19^) to determine the proper proportion *q*. In fact, this result indicates how to modify the data set to reflect the misclassification costs optimally: if we modify the distribution of the class variable *V* on the data set to a new one, say $({\widetilde{p}}_+,\,{\widetilde{p}}_-)$, multiplying any of the components of the original distribution $(p_+,\,p_-)$ by a constant proportional to the associated misclassification costs, the resulting distribution has the following property: *choose the classifier that minimizes misclassification error rates (maximizes accuracy) under the new distribution is equivalent to choosing the classifier that minimizes the expected cost under the original distribution*.

The rationale behind this theorem is as follows: consider a probabilistic classifier learned from the modified data set. Given a new instance, if the classifier assigns it to the positive class, the expected cost (with respect to the class distribution of the original data set) is: $0 \times p_+ + c_-\, p_-=c_-\,p_-$. Similarly, if the classifier assigns it to the negative class, the expected cost is $0\times p_- + c_+\,p_+=c_+ \, p_+$. Then, the assigned class that minimizes the expected cost (with respect to the class distribution of the original data set) is

$$\begin{aligned} {\left\{ \begin{array}{ll} + &{} \mathrm{if } \,\,c_-\,p_- < c_+\,p_+\\ - &{} \mathrm{if } \,\, c_-\,p_- > c_+\,p_+, \end{array}\right. } \end{aligned}$$

which matches the class that minimizes misclassification error rates under the new distribution, which are ${\widetilde{p}}_-$ if the predicted class is “+”, and ${\widetilde{p}}_+$ if the predicted class is “−”, provided that

$$\begin{aligned} {\widetilde{p}}_+ = C\,p_+\,c_+ \quad \text {and}\quad {\widetilde{p}}_- = C\,p_-\,c_- \end{aligned}$$

for some constant $C>0$. Since ${\widetilde{p}}_+$ and ${\widetilde{p}}_-$ must add up to 1, we obtain that the constant necessarily has to be

$$\begin{aligned} C=\frac{1}{p_+\,c_+ + p_-\,c_-}\,. \end{aligned}$$

Therefore, to account for misclassification costs in the sampling indirect method for cost-sensitive learning, we will enlarge the original data set by over-sampling the minority class, and by the Folk Theorem we will choose the proportion *q* that the minority class will represent in the enlarged data set such that the modified distribution of the class variable is

$$\begin{aligned} {\widetilde{p}}_+ = \frac{p_+\,c_+}{p_+\,c_+ + p_-\,c_-}\quad \mathrm{and} \quad {\widetilde{p}}_- = \frac{p_-\,c_-}{p_+\,c_+ + p_-\,c_-} \end{aligned}$$

That is,

2 $$\begin{aligned} q&={\widetilde{p}}_+=\frac{p_+\,c_+}{p_+\,c_+ + p_-\,c_-}= \frac{p_+\,\gamma }{p_+\,\gamma + p_-}=\frac{m_+\,\gamma }{m_+\,\gamma + m_-} \end{aligned}$$

with $\gamma =\frac{c_+}{c_-}$, showing the functional dependence of *q* on the initial number of instances of each class and on the misclassification costs rate $\gamma$.

*Remark 1:* Note that by (2), $q \in (\frac{m_+}{M},\,1)$. Indeed, since $m_+\,\gamma + m_- >m_+\,\gamma$ we have that $q<1$. On the other hand, using $M=m_+ + m_-$,

$$\begin{aligned} q&=\frac{m_+\,\gamma }{m_+\,\gamma + m_-}>\frac{m_+}{M} \Longleftrightarrow \gamma \,(m_+ + m_-)> m_+\,\gamma + m_- \Longleftrightarrow \gamma >1\,, \end{aligned}$$

which is true by assumption.

## Experiments

We have performed some experiments to evaluate BOSME and compare it to the benchmark SMOTE. For that, we consider different open access data sets and some state-of-the-art classifiers. In addition to SMOTE, we also compare BOSME with the over-sampling method ROSE^20^, which is based on a smoothed bootstrap form of re-sampling from data, used to draw artificial samples from the feature space neighborhood around the minority class using a probability distribution centered at a randomly selected case and based on a smoothing matrix of scale parameters.

### Data sets

In the experimentation phase, the data sets summarized in Tables 1 and 2 were considered, some with only categorical features, others with mixed features (categorical and continuous), and the rest with all the features continuous. In the data preprocessing phase, the missing cases of the categorical variables have been consigned as a new category different from the others, while for the continuous variables they have been eliminated. Also, when the class variable originally had more than two categories, it has been made binary by category merging.

**Table 1:** Summary of data sets. For the data set **Car evaluation**, the categories good and v-good on one side, and acc and unaccc on the other, were merged. For **Solar flare**, we have taken the second data section flare.data2 as the data set as it seems to be more reliable. In **Pizza price**, *extra-cheese* has been taken as output class variable.

Data set	Repository	Instances	Minority class	Majority class	Categorical features	Continuous features
Car evaluation	UCI	1728	134 (7.75%)	1594 (92.25%)	6	0
Spect heart	UCI	267	55 (20.6%)	212 (79.4%)	22	0
Balance scale	UCI	625	49 (7.84%)	576 (92.16%)	4	0
Monks	UCI	415	186 (44.82%)	229 (55.18%)	6	0
Post-operative patient	UCI	88	24 (27.27%)	64 (72.73%)	8	0
Tic-tac-toe endgame	UCI	958	332 (34.66%)	626 (65.34%)	9	0
Solar Flare	UCI	1066	43 (4.03%)	1023 (95.97%)	11	0
Breast cancer	UCI	286	85 (29.72%)	201 (70.28%)	9	0
Pizza price	KAGGLE	129	43 (33.33%)	86 (66.67%)	4	2
Haberman	KEEL	306	81 (26.47%)	225 (73.53%)	0	3
Saheart	KEEL	462	160 (34.63%)	302 (65.37%)	1	8
Happiness	UCI	143	66 (46.15%)	77 (53.85%)	6	0

**Table 2:** Summary of data sets whose results are statistically insignificant to compare BOSME with SMOTE.

Data set	Repository	Instances	Minority class	Majority class	Categorical features	Continuous features
Congresional Voting	UCI	435	168 (38.62%)	267 (61.38%)	16	0
Lymphography	KEEL	148	6 (4.05%)	142 (95.95%)	18	0
Diabetes	UCI	520	200 (38.46%)	320 (61.54%)	15	0
Zoo	UCI	101	5 (4.95%)	96 (95.05%)	16	0
Qualitative Bankrupcity	UCI	250	107 (42.8%)	143 (57.2%)	6	0
Dishonest	UCI	322	97 (30.12%)	225 (69.88%)	4	0
Lung	KAGGLE	309	39 (12.62%)	270 (87.38%)	14	1
Indian	KAGGLE	399	195 (48.87%)	204 (51.13%)	5	0
TAE	UCI	151	29 (19.21%)	122 (80.79%)	2	3
Bupa	KEEL	345	145 (42.03%)	200 (57.97%)	0	6

### Classifiers

In the experiments, we used the following three supervised machine learning algorithms for classification to compare BOSME with SMOTE and ROSE.

1. *Logistic Regression* (LR) is a Supervised Machine Learning method dedicated to classification tasks that has gained popularity during the last two decades, especially in the financial sector. This method uses a linear regression equation to produce discrete binary outputs. We implement it through the R function stats::glm, using the argument “family $=$ binomial” (see^21^). Note that stats package is a part of R (R Core Team (2020). R: A language and environment for statistical computing. R Foundation for Statistical Computing, Vienna, Austria. URL https://www.R-project.org/.)
2. *Random Forest* (RF)^22^ is an ensemble learning method for classification, based on a series of decision trees as basic classifiers (we use the default 500). The output is the mode of the classes of the individual trees (according to the majority vote criterion). We use its implementation in the mlearning R package (see “R packages references” at end of paper).
3. *Support Vector Machine* (SVM)^23^ uses a representation of the instances of the data set by mapping them as points in a space, in such a way that they are separated in the two categories by a gap as wide as possible. A new instance is then mapped into this space; depending on which side of the gap its point representation falls on, the instance class is predicted to be one or the other. We use the radial basis function (RBF) kernel to define the map, and the implementation of the algorithm in the mlearning R package (see “R packages references” at end of paper).

### Implementation

**Figure 2:**
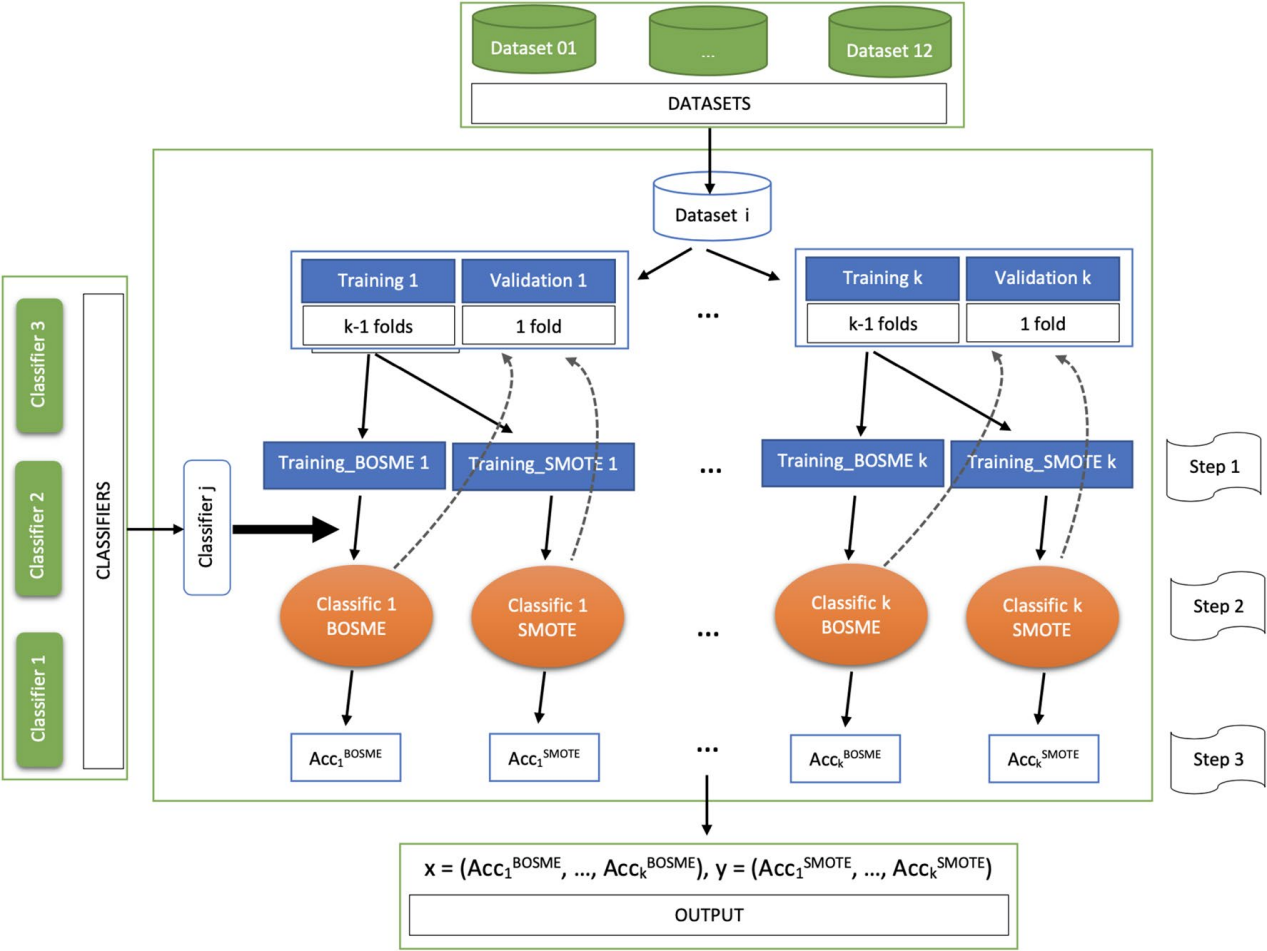
Implementation: Stage 1 architecture to compare BOSME with SMOTE (analogous with ROSE).

The implementation of the experimental phase has been carried out in two stages. In Stage 1, for any of the data sets described in “Section Data sets”, since we will be using *k*-fold cross-validation (with $k=10$), we first randomly divide the data set into *k* folds of rougly the same dimension, and for any fold, we reserve it for later use as a validation set, and use the rest as a training set. Then, for any pair of training/validation sets, we follow the steps below (see the architecture of the proposed implementation in Fig. 2).

Step 1: Use the BOSME over-sampling technique (Algorithm 1), as well as SMOTE and ROSE, for cost-sensitive learning of the classifiers from the initial training data set, using misclassification costs and the initial distribution of the class variable. That is, determine the proportion *q* that the minority class “+” should represent in the enlarged training set by (2), and apply the over-sampling technique to obtain it. To learn the Bayesian network in Algorithm 1, we use the hill-climbing algorithm implemented in the R package bnlearn by means of the function hc. As score we use the option loglik when all features are categorical, loglik-cg in the conditionally Gaussian mixed Bayesian network case, and loglik-g in the Gaussian case with all the features continuous. For the simulation of the new instances corresponding to the minority class from the Bayesian network with the LS algorithm, we use the rbn function from the bnlearn package. We use the implementation of SMOTE given by the smote function of the R package performanceEstimation, and function ROSE of the R package of the same name, for the implementation of the ROSE oversampling method. See “R packages references” at the end of the paper.
Step 2: Learn the classifiers introduced in “Section Classifiers” from the enlarged training data sets, obtained in Step 1, with BOSME, SMOTE and ROSE for comparison.
Step 3: Evaluate the classifiers using the original validation set. As performance metric we use accuracy, as explained in “Section Application: sampling indirect method for cost-sensitive learning”.

Therefore, for each data set we get a 10-dimensional vector of values for any classifier and any of the over-sampling methods as output of Stage 1 (see the output in the scheme depicted in Fig. 2).

**Figure 3:**
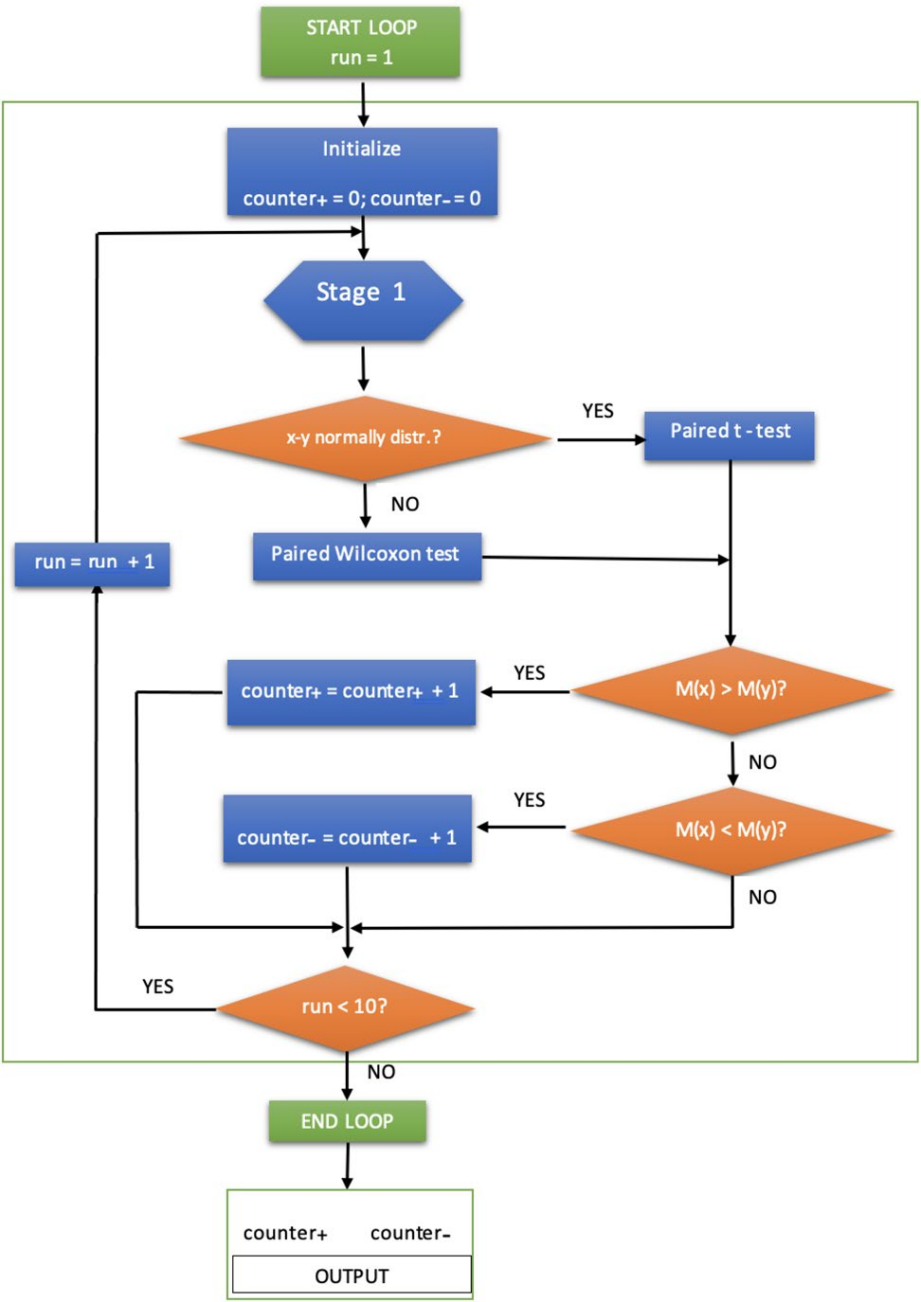
Implementation: Stage 2 procedure flowchart covering the Stage 1 architecture shown in Fig. 2. The letter “M” in the decision boxes denotes the mean/average or the median, depending on whether or not the normality of the distribution of the sample $x-y$ can be assumed, respectively, where *x* and *y* are the output of Stage 1 (Fig. 2).

To avoid possible bias, in a second stage we repeat the described procedure 10 runs with different seeds for the random splitting of the data set into the *k* folds. Next, we analyze the results obtained to make comparisons between BOSME, SMOTE and ROSE as shown in the flowchart described in Fig. 3, which encompasses the architecture portrayed in Fig. 2.

For that, we have performed pairwise statistical tests of hypotheses to determine the significance of the results. More specifically, for any data set and for any run, given the classifier, we can perform a paired test to compare the mean (or median, as appropriate) accuracy of the two corresponding samples of size 10 obtained using the BOSME, SMOTE and ROSE over-sampling methods. We use the Shapiro-Wilk normality test to choose between the parametric t-test and the paired non-parametric Wilcoxon signed-rank test, with the criterium that if its *p* value is $<0.05$, normality cannot be assumed and therefore the last one must be carried out; otherwise, we can use the paired t-test.

In this way, for each data set, each classifier and each over-sampling method, we have two counters that collect the number of runs, of the 10, in which BOSME outperforms the other over-sampling method (counter$_+$) and the number of runs for which just the opposite happens (counter$_-$), which are shown as the output of the procedure described in Fig. 3. The above procedure is performed for different values of the cost ratio $\gamma =\frac{c_+}{c_-}$, varying between 5 and 50, from 5 to 5. The results obtained are explained in the next section.

## Results

### BOSME versus SMOTE

Tables 8, 9, 10, 11 and 12 in the "Appendix" summarize the results of the experimental process when comparing BOSME with SMOTE. They record the number of runs (out of 10 possible) for which there is statistical evidence in favor of BOSME (positive number, counter$$_+$$) and in favor of SMOTE (negative number, counter$$_-$$). If only a positive number appears in a box, it means that counter$$_- = 0$$, and the same happens if only a negative number appears, which means that counter$$_ + = 0$$. As usual, $$^{*}$$ means statistical significance at the 0.05 level, $$^{**}$$ at 0.01, and $$^{***}$$ at 0.001.

The corresponding exact Binomial *p* values (used instead of the McNemar test, because the sample is small) have also been recorded in these tables, provided that they are significant ($<0.05$), for any data set and classifier, for the different values of $\gamma$. For example, in Table 8 with $\gamma =10$, for the SVM classifier and the Post operative data set, counter$_+=9$ and counter$_-=0$, that is, there are 9 of the 10 runs with statistical differences between BOSME and SMOTE, all in favor of BOSME, with a one-sided *p* value for the exact Binomial test equal to

$$\begin{aligned} P(B(n,0.5)=\text{ counter}_+)&= P(B(9,0.5)=9) =\left( {\begin{array}{c}9\\ 9\end{array}}\right) \big (\frac{1}{2}\big )^9\big (\frac{1}{2}\big )^0= 0.001953125^{**} \end{aligned}$$

One more example: in the same table, for the data set Flare with the SVM classifier, counter$_+=8$ and counter$_-=1$, which means that out of 10 runs, there are 9 with statistical differences between BOSME and SMOTE, 8 in favor of BOSME and 1 in favor of SMOTE, giving a one-sided *p* value for the exact Binomial test in favor of BOSME equal to

$$\begin{aligned} P(B(n,0.5)=\text{ counter}_+)&= P(B(9,0.5)=8)=\left( {\begin{array}{c}9\\ 8\end{array}}\right) \big (\frac{1}{2}\big )^8\big (\frac{1}{2}\big )^1= 0.01757812^{*} \end{aligned}$$

**Figure 4:**
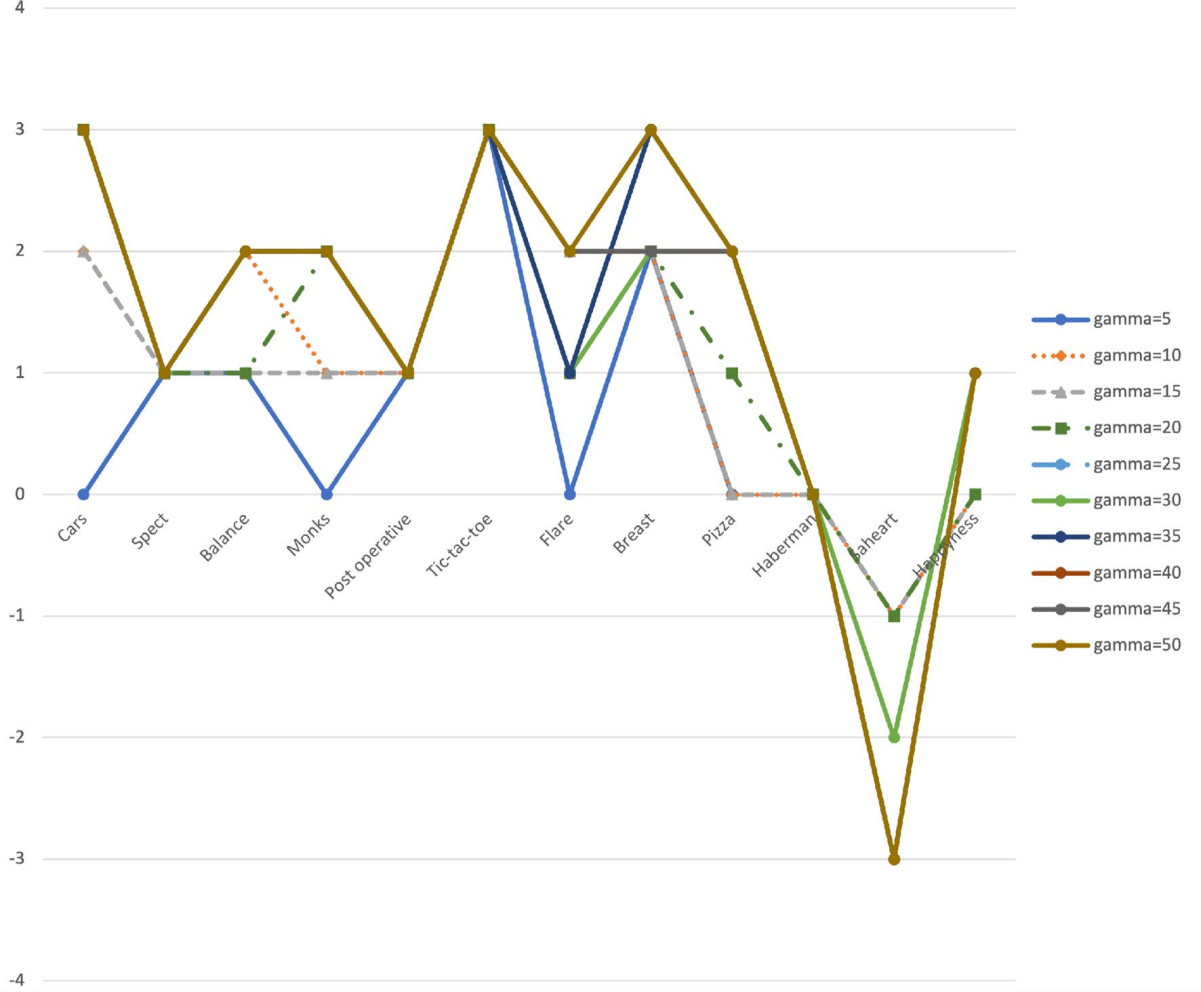
Representation of the information in the Table 3, by data set, for the different values of $\gamma$.

In Table 3 we summarize by data set the results given in Tables 8, 9, 10, 11 and 12, showing with a positive number for how many classifiers BOSME has been statistically successful against SMOTE (*p* value $<0.05$ and counter$_+>$counter$_-$). On the contrary, a negative number expresses the number of classifiers with which SMOTE has been significantly better than BOSME (*p* value $<0.05$ and counter$_->$counter$_+$). Since we have used 3 different classifiers, $+3/-3$ are the best and worst ratings, respectively, in favor of BOSME. The white boxes do not show significant results in any sense. The information from Table 3 is represented in Fig. 4, where we can observe the behavior of BOSME with respect to SMOTE for the different values of the cost ratio $\gamma$ and any data set. As expected, although this behavior varies with the data set, in all the cases except the Saheart data set, BOSME outperforms SMOTE, especially for high values of $\gamma$. In Table 3 we also record the value of the $\beta$-score, which we enter as the sums per column. So $\beta$ ranges from $-36$ to $+36$ ($36=12\times 3$, with 12 data sets and 3 classifiers).

**Table 3:** Summary of the results, by data set, for different values of the cost ratio $\gamma =\frac{c_+}{c_-}$. The numbers in the boxes indicate for how many classifiers, of the possible 3, there is statistical significance in favor of BOSME (positive) or in favor of SMOTE (negative, in bold). For each $\gamma$, we take count of the $\beta$-score.

$\gamma$	5	10	15	20	25	30	35	40	45	50
Car eval.		+2	+2	+3	+3	+3	+3	+3	+3	+3
Spect heart	+1	+1	+1	+1	+1	+1	+1	+1	+1	+1
Balance	+1	+2	+2/**−1**	+2/**−1**	+2	+2	+2	+2	+2	+2
Monks		+1	+1	+2	+2	+2	+2	+2	+2	+2
Post-oper.	+1	+1	+1	+1	+1	+1	+1	+1	+1	+1
Tic-tac-toe	+3	+3	+3	+3	+3	+3	+3	+3	+3	+3
Solar flare		+2	+2	+1	+1	+1	+1	+2	+2	+2
Breast	+2	+2	+2	+2	+2	+2	+3	+2	+2	+3
Pizza				+1	+2	+2	+2	+2	+2	+2
Haberman	+1/**−1**	+1/**−1**	+1/**−1**	+1/**−1**	+1/**−1**	+1/**−1**	+1/**−1**	+1/**−1**	+1/**−1**	+1/**−1**
Saheart		/**−1**	/**−1**	/**−1**	/**−3**	/**−2**	/**−3**	/**−3**	/**−3**	/**−3**
Happiness					+1	+1	+1	+1	+1	+1
$\beta$-score	+8	+13	+12	+14	+15	+16	+16	+16	+16	+17

Both in Table 3 and in Fig. 4 the data sets “Haberman” and “Saheart” appear to behave differently of the rest. These data sets have a characteristic that, together with “Pizza”, differentiates them from the rest, and that is the fact that some of the features are continuous, so the Bayesian network that is learned in the BOSME method is no longer a standard but a Gaussian Bayesian network (“Haberman”) or a hybrid Bayesian network (“Saheart”, “Pizza”). Therefore, it is not surprising that with these datasets BOSME does not behave so well with respect to SMOTE, since this last method has been designed for datasets with continuous features, and although SMOTE can also be used with categorical features, seems that in this case BOSME outperforms it. For both “Haberman” and “Saheart”, all the features (in the first), or almost (8 of 9 in the second), are continuous. However, “Pizza” is a hybrid case in which of the 6 features, only 2 are continuous, behaving more in line with the rest of the datasets.

**Figure 5:**
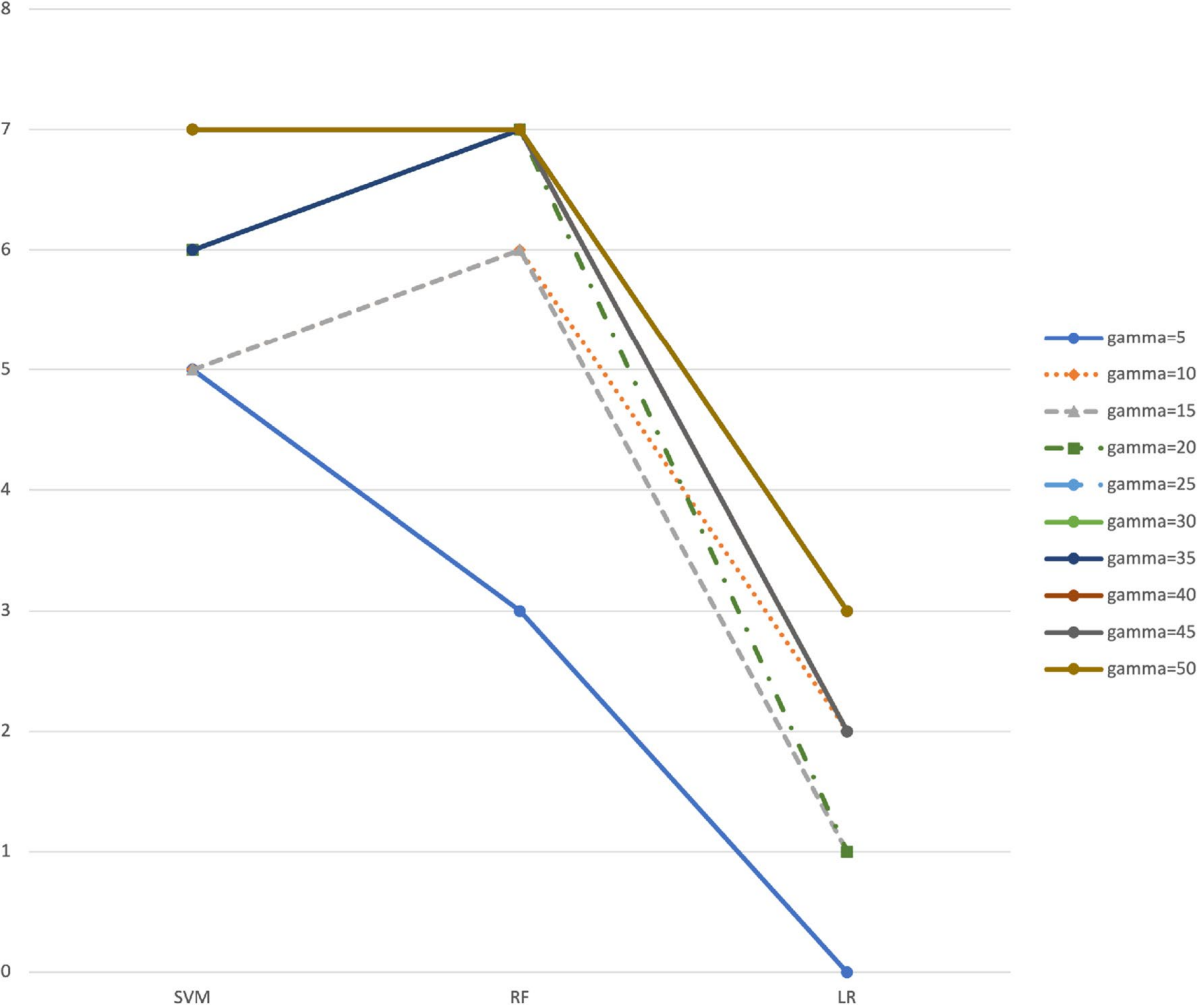
Graphic representation of the information in Table 4, by type of classifier, for the different values of $\gamma$.

Table 4 below is complementary to Table 3 in summarizing by classifier the results given in Tables 8, 9, 10, 11 and 12. Since we have considered 12 different data sets for which there are significant results, $+12/-12$ are the best and worst ratings, respectively, in favor of BOSME. Figure 5 below represents the information in this table and allows comparing the behavior of BOSME with respect to SMOTE for the different values of the cost ratio $\gamma$ and any type of classifier. We see in Fig. 5 that although with some classifiers the behavior of BOSME relative to that of SMOTE is better than with others (it seems that with the Logistic Regression it is clearly worse), in general it improves when $\gamma$ increases, regardless of the chosen classifier.

**Table 4:** Summary of the results, by classifier, for different values of the cost ratio $\gamma =\frac{c_+}{c_-}$. The numbers in the boxes indicate for how many data sets, of the possible 12, there is statistical significance in favor of BOSME (positive) or in favor of SMOTE (negative, in bold).

$\gamma$	5	10	15	20	25	30	35	40	45	50
SVM	+5	+7/**−2**	+7/**−2**	+8/**−2**	+8/**−2**	+8/**−2**	+8/**−2**	+9/**−2**	+9/**−2**	+9/**−2**
RF	+3	+6	+6	+7	+8/**−1**	+8/**−1**	+8/**−1**	+8/**−1**	+8/**−1**	+8/**−1**
LR	+1/**−1**	+2	+2/**−1**	+2/**−1**	+3/**−1**	+3	+4/**−1**	+3/**−1**	+3/**−1**	+4/**−1**

**Figure 6:**
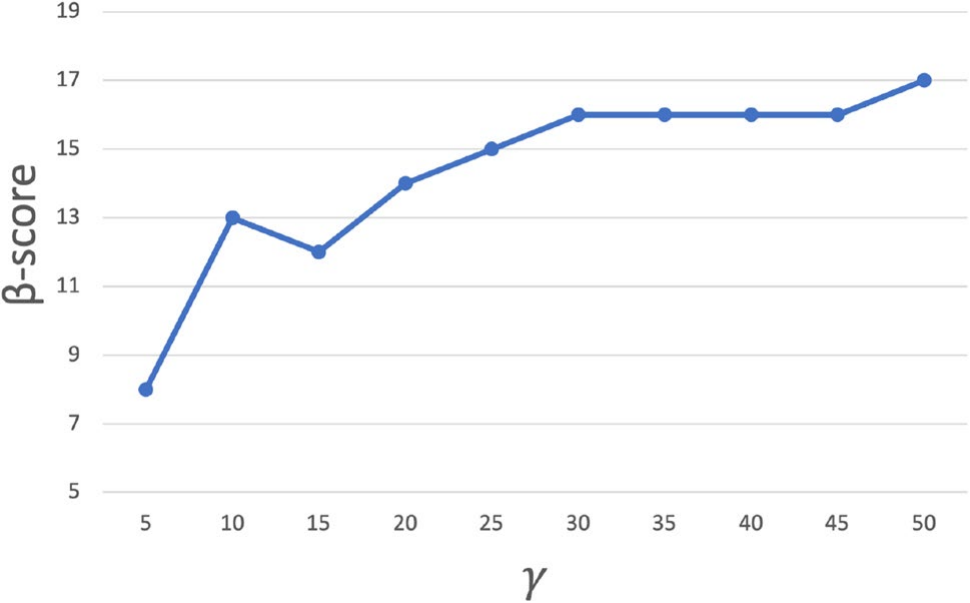
Graphic representation of the $\beta$-score for the results of Table 3, where the evolution as the cost ratio $\gamma$ increases can be observed.

We can represent the $\beta$-score provided by Table 3 with the help of the graph in Fig. 6, where we can observe two interesting results: (a) the $\beta$-score turns out to be always positive, and (b) it increases with the cost ratio $\gamma$.

We can perform some statistical tests of hypotheses to check the importance of these two observed phenomena.

- **Positiveness of the **$\beta$-**score:** Indeed, in Table 3, of the 10 considered values of $\gamma$, the number of them for which the corresponding $\beta$-score is strictly positive is 10. The corresponding *p* value for the exact Binomial test is
  $$\begin{aligned} P(B(n=10,\,p=0.5)=10)=\left( {\begin{array}{c}10\\ 10\end{array}}\right) \,\big (\frac{1}{2}\big )^{10}\,\big (\frac{1}{2}\big )^0 = 0.0009765625^{***}\,, \end{aligned}$$
  which implies a statistical significance in favor of BOSME (the one associated with the positive value of the $\beta$-score).
- **Trend monotonicity of the **$\beta$-**score with respect to **$\gamma$: We observe in Table 3 that, in general, the values of the $\beta$-score increase with $\gamma$ (see Fig. 6). To check the statistical significance of this trend monotonicity, we use the Mann–Kendall test^24,25^, which statistically evaluates whether there is a monotonic upward or downward trend of the variable of interest, which is the $\beta$-score, relative to an ordered variable like $\gamma$ (which does not necessarily have to be temporary in nature). A monotone up (down) trend means that the variable consistently increases (decreases) as $\gamma$ increases. If the Mann–Kendall test gives a significant positive or negative trend (*p* value $<0.05$), which in this case will be positive, Sen’s slope captures the magnitude of that trend (that is, it provides an estimate of the average increase in the $\beta$-score per increase of a section of $\gamma$). The results of the Mann–Kendall test, and also Spearman’s rank correlation test, are given in Table 5 below.

**Table 5:** $\beta$-score. Mann–Kendall test: $\tau$ statistic, two-sided *p* value and Sen’s slope with a confidence interval (CI) of $95\%$. Spearman’s rank correlation test: rho statistic and one-sided *p* value for the alternative hypothesis that $\rho >0$.

Mann–Kendall			Spearman’s rank correlation	
$\tau$	Two-sided *p* value	Sen’s slope	$\rho$	*p* value
0.649	0.000091***	1.34286, 95% CI: $(0.4,\,2.5)$	0.95672	0.000007***

Table 5 shows that there is indeed a significant monotonically increasing trend in the $\beta$-score, as the cost ratio $\gamma$ increases, which is associated with a better behavior of BOSME with respect to SMOTE. The empirical evidence is in the sense that: BOSME outperforms SMOTE for all the values tested in the experimental phase, but it also does so more the higher the value of the cost ratio $\gamma$.

#### BOSME versus ROSE

Comparing BOSME with ROSE similarly to the comparison with SMOTE, we find that there are significant differences only for the 3 data sets: Pizza price, Haberman, and Saheart. The results are in Tables 6 and 7.

**Table 6:** Summary of the results, by data set, for different values of the cost ratio $\gamma =\frac{c_+}{c_-}$. The numbers in the boxes indicate for how many classifiers, of the possible 3, there is statistical significance in favor of BOSME (positive) or in favor of ROSE (negative, in bold). For each value of $\gamma$, we take count of the $\beta$-score.

$\gamma$	5	10	15	20	25	30	35	40	45	50
Pizza		/**−1**	/**−1**	/**−1**						
Haberman		+1	+1	+1						
Saheart	+2	+2	+2	+2	+2	+2	+2	+2	+3	+2
$\beta$-score	+2	+2	+2	+2	+2	+2	+2	+2	+3	+2

**Table 7:** Summary of the results, by classifier, for different values of the cost ratio $\gamma =\frac{c_+}{c_-}$. The numbers in the boxes indicate for how many data sets, of the possible 3, there is statistical significance in favor of BOSME (positive) or in favor of ROSE (negative, in bold).

$\gamma$	5	10	15	20	25	30	35	40	45	50
SVM		+1/**−1**	+2/**−1**	+2/**−1**	+1	+1	+1	+1	+1	+1
RF	+1	+1							+1	
LR	+1	+1	+1	+1	+1	+1	+1	+1	+1	+1

**Positiveness of the **$\beta$-**score for BOSME versus ROSE:** in Table 6 we observe that of the 10 values considered for $\gamma$, the number of them for which the corresponding $\beta$-score is strictly positive is 10. The corresponding *p* value for the exact Binomial test is the same as when compared to SMOTE: 0.0009765625*** in favor of BOSME. Since, except in one case, all values of the $\beta$-score are constant with $\gamma$, there is not statistical significance for trend monotonicity (two-sided Mann–Kendall *p* value 0.1616).

## Conclusion

The introduced BOSME is an over-sampling method that has achieved moderate to good performance against the SMOTE and ROSE over-sampling methods, through a series of experiments, in the context of the indirect cost-sensitive learning approach. This approach consists of enlarge the original imbalanced data set with a number of artificially generated minority instances, which is determined from the misclassification costs. In this way, we use over-sampling methods and take misclassification costs into account, to extend the data used to feed cost-insensitive supervised learning algorithms.

In fact, the results empirically show that in the context of the *cost-sensitive* approach,

- there is statistical evidence in favor of BOSME dominance over SMOTE,
- this evidence is stronger as the cost ratio $\gamma$ increases, and for data sets with all categorical features (above continuous or mixed type),
- there is slight evidence in favor of BOSME’s dominance over ROSE, which remains constant as $\gamma$ varies.

Other highlights of this new method that distinguish it from SMOTE are:

1. BOSME is a novel *over-sampling* method based on a new paradigm, using Bayesian networks.
2. The generation of the artificial instances of the minority class is carried out from a model for the relationship between the features, instead of using the idea of distance between instances, which is the paradigm followed by SMOTE and its derivatives.
3. Maximizing the likelihood function is the criterion for choosing the Bayesian network to use as a model. In this way, the model will be the most plausible given the minority instances, and approximates their probability distribution.
4. The Bayesian network is then a good model that captures the relationship between the features for the minority class, with which generate new instances of this class that are really representative, and from them, learn classifiers that can better differentiate between the two classes, improving their predictive power.
5. This method has wide applicability, for all kinds of features.

As a consequence, we conclude that BOSME, which is the method presented in this paper, is a reasonable over-sampling method that has shown very promising results for implementing indirect cost-sensitive learning, in the duel against the benchmark SMOTE, especially in the case of having data sets with all features of categorical type, and for a moderate to high cost ratio. In the case of data sets with mixed features, BOSME does not perform better but it can withstand SMOTE’s onslaught. With respect to ROSE, significant differences are only observed, in favor of BOSME, in the case of mixed features. Therefore, given that its results in the experimental phase have been very promising, we promote the use of BOSME as an over-sampling methodology with completely general applicability.

In future research, we will try to deepen the study of the effect of the type of features and the distribution of the class variable in the data set, on the behavior of BOSME, and we will compare it with other methods of over-sampling using more data sets. We are also interested in considering extensions/modifications of the version of BOSME that we present in this paper, for example by introducing tree-width constraints on the learning structure that would lead to less complex structures.

## R packages references

- performanceEstimation (version 1.1.0). Function: smote. Reference: Torgo, L. An Infra-Structure for Performance Estimation and Experimental Comparison of Predictive Models in R (2014). arXiv:1412.0436 [cs.MS]
- ROSE (version 0.0-3). Function: ROSE. Reference: Lunardon, N., Menardi, G.,Torelli, N. ROSE: a Package for Binary Imbalanced Learning. R Journal, 6:82–92 (2014).
- mlearning (version 1.0-0). Functions: mlSvm and mlRforest. Reference: Grosjean, Ph., Denis, K. mlearning: Machine learning algorithms with unified interface and confusion matrices (2013). https://CRAN.R-project.org/package=mlearning
- bnlearn (version 4.7). Functions: hc and rbn. Reference: Scutari, M. Learning Bayesian Networks with the bnlearn R Package. Journal of Statistical Software vol. 35(3), pp. 1–22 (2010). http://www.jstatsoft.org/v35/i03/

## Data Availability

The data sets analyzed during the current study are available in the following repositories: (1) UCI: https://archive.ics.uci.edu/, (2) KAGGLE: https://www.kaggle.com/, (3) KEEL https://sci2s.ugr.es/keel/data sets.php.

## Appendix

See Tables 8, 9, 10, 11 and 12.

**Table 8:** Number of runs (of the possible 10) for which there is statistical evidence in favor of BOSME (positive number, counter$_+$) or SMOTE (negative in bold, counter$_-$), and the corresponding exact Binomial *p* value, for any data set with significant differences, and classifier: Support Vector Machine (SVM), Random Forest (RF) and Logistic Regression (LR). $\gamma =5,\,10$.

	$\gamma =5$			$\gamma =10$		
	SVM	RF	LR	SVM	RF	LR
Car eval.				+10 0.00098***		+7 0.00781**
Spect heart	+6 0.01563*			+6 0.01563*		
Balance	+10 0.00098***			+10 0.00098***	+10 0.00098***	
Monks					+6 0.01563*	
Post-oper.	+7 0.00781**			+9 0.00195**		
Tic-tac-toe	+10 0.00098***	+10 0.00098***	+10 0.00098***	+10 0.00098***	+10 0.00098***	+10 0.00098***
Solar flare				+8/**−1** 0.01758*	+7 0.00781**	
Breast	+9 0.00195**	+10 0.00098***		+10 0.00098***	+10 0.00098***	
Pizza				+10 0.00098***		
Haberman		+5 0.03125*	/**−6** **0.01563***	/**−9** **0.00195****	+7 0.00781**	
Saheart				/**−7** **0.00781****		
Happiness

**Table 9:** Analogous to Table 8 but with $\gamma =15,\,20$.

	$\gamma =15$			$\gamma =20$		
	SVM	RF	LR	SVM	RF	LR
Car eval.	+10 0.00098***		+10 0.00098***	+10 0.00098***	+7 0.00781**	+10 0.00098***
Spect heart	+6 0.01563*			+6 0.01563*		
Balance	+10 0.00098***	+10 0.00098***	**−10** **0.00098*****	+10 0.00098***	+10 0.00098***	**−9** **0.00195****
Monks		+10 0.00098***		+5 0.03125*	+9 0.00195**	
Post-oper.	+9 0.00195**			+9 0.00195**		
Tic-tac-toe	+10 0.00098***	+10 0.00098***	+10 0.00098***	+10 0.00098***	+10 0.00098***	+10 0.00098***
Solar flare	+6 0.01563*	+8 0.00391**			+8 0.00391**	
Breast	+10 0.00098***	+10 0.00098***		+10 0.00098***	+10 0.00098***	
Pizza				+8 0.00391**		
Haberman	/**−10** **0.00098*****	+10 0.00098***		/**−10** **0.00098*****	+10 0.00098***	
Saheart	/**−10** **0.00098*****			/**−10** **0.00098*****		
Happiness

**Table 10:** Analogous to Table 8 but with $\gamma =25,\,30$.

	$\gamma =25$			$\gamma =30$		
	SVM	RF	LR	SVM	RF	LR
Car eval.	+10 0.00098***	+10 0.00098***	+10 0.00098***	+10 0.00098***	+8 0.00391**	+10 0.00098***
Spect heart	+6 0.01563*			+6 0.01563*		
Balance	+10 0.00098***	+10 0.00098***		+10 0.00098***	+10 0.00098***	
Monks	+6 0.01563*	+10 0.00098***		+9 0.00195**	+10 0.00098***	
Post-oper.	+9 0.00195**			+9 0.00195**		
Tic-tac-toe	+10 0.00098***	+10 0.00098***	+10 0.00098***	+10 0.00098***	+10 0.00098***	+10 0.00098***
Solar flare		+8 0.00391**			+9 0.00195**	
Breast	+10 0.00098***	+10 0.00098***		+10 0.00098***	+9 0.00195**	
Pizza	+7 0.00781**		+5 0.03125*	+10 0.00098***		+6 0.01563*
Haberman	/**−10** **0.00098*****	+10 0.00098***		/**−10** **0.00098*****	+10 0.00098***	
Saheart	/**−10** **0.00098*****	/**−5** **0.03125***	/**−6** /**0.01563***	/**−10** **0.00098*****	/**−7** **0.00781****	
Happiness		+5 0.03125*			+6 0.01563*

**Table 11:** Analogous to Table 8 but with $\gamma =35,\,40$.

	$\gamma =35$			$\gamma =40$		
	SVM	RF	LR	SVM	RF	LR
Car eval.	+10 0.00098***	+10 0.00098***	+10 0.00098***	+10 0.00098***	+10 0.00098***	+10 0.00098***
Spect heart	+6 0.01563*			+6 0.01563*		
Balance	+10 0.00098***	+10 0.00098***		+10 0.00098***	+10 0.00098***	
Monks	+10 0.00098***	+10 0.00098***		+7 0.00781**	+10 0.00098***	
Post-oper.	+10 0.00098***			+10 0.00098***		
Tic-tac-toe	+10 0.00098***	+10 0.00098***	+10 0.00098***	+10 0.00098***	+10 0.00098***	+10 0.00098***
Solar flare		+9 0.00195**		+5 0.03125*	+9 0.00195**	
Breast	+10 0.00098***	+10 0.00098***	+6 0.01563*	+10 0.00098***	+10 0.00098***	
Pizza	+9 0.00195**		+6 0.01563*	+8 0.00391**		+5 0.03125*
Haberman	/**−10** **0.00098*****	+10 0.00098***		/**−10** **0.00098*****	+10 0.00098***	
Saheart	/**−10** **0.00098*****	/**−9** **0.00195****	/**−6** /**0.01563***	/**−10** **0.00098*****	/**−9** **0.00195****	/**−8** /**0.00391****
Happiness		+7 0.00781**			+7 0.00781**

**Table 12:** Analogous to Table 8 but with $\gamma =45,\,50$.

	$\gamma =45$			$\gamma =50$		
	SVM	RF	LR	SVM	RF	LR
Car eval.	+10 0.00098***	+10 0.00098***	+10 0.00098***	+10 0.00098***	+10 0.00098***	+10 0.00098***
Spect heart	+6 0.01563*			+6 0.01563*		
Balance	+10 0.00098***	+10 0.00098***		+10 0.00098***	+10 0.00098***	
Monks	+9 0.00195**	+10 0.00098***		+10 0.00098***	+10 0.00098***	
Post-oper.	+10 0.00098***			+10 0.00098***		
Tic-tac-toe	+10 0.00098***	+10 0.00098***	+10 0.00098***	+10 0.00098***	+10 0.00098***	+10 0.00098***
Solar flare	+7 0.00781**	+9 0.00195**		+7 0.00781**	+9 0.00195**	
Breast	+10 0.00098***	+10 0.00098***		+10 0.00098***	+9 0.00195**	+5 0.03125*
Pizza	+10 0.00098***		+7 0.00781**	+10 0.00098***		+6 0.01563*
Haberman	/**−10** **0.00098*****	+10 0.00098***		/**−10** **0.00098*****	+10 0.00098***	
Saheart	/**−10** **0.00098*****	/**−8** **0.00391****	/**−9** /**0.00195****	/**−10** **0.00098*****	/**−8** **0.00391****	/**−9** /**0.00195****
Happiness		+8 0.00391**			+7 0.00781**	

## Abbreviations

BOSME: Bayesian network-based over-sampling method
SMOTE: Synthetic minority over-sampling technique
SMOTE-NC: Synthetic minority over-sampling technique-nominal continuous
SVDD: Support vector data description
G-SMOTE: Variant of SMOTE that allows the generation of synthetic instances in a geometric region around the selected instances
BN: Bayesian network
DAG: Directed acyclic graph (the graphical part of a BN)
PA: Set of nodes that are parents, in the DAG, of a given node
MLE: Maximum likelihood estimation method for parameters estimation
LS: Logic Sampling algorithm
ROSE: Random over-sampling examples
LR: Logistic regression
RF: Random forest
SVM: Support vector machine
RBF: Radial basis function kernel

## Data availability

The R source code for this project is available upon reasonable request.
